# Heme in Bacterial Pathogenesis and as an Antimicrobial Target

**DOI:** 10.1021/acs.chemrev.5c00528

**Published:** 2025-11-17

**Authors:** Pei-Yi Chen, Eric P. Skaar

**Affiliations:** † Department of Pathology, Microbiology, and Immunology, Vanderbilt University, Nashville, Tennessee 37232, United States; ‡ Vanderbilt Institute for Infection, Immunology, and Inflammation, 12328 Vanderbilt University Medical Center, Nashville, Tennessee 37232, United States

## Abstract

Heme is an essential molecule required for critical biochemical processes in most vertebrates and bacteria. During infections, vertebrate hosts sequester heme away from invading pathogens, a process known as nutritional immunity, driving bacteria to evolve diverse mechanisms to evade this immunity and cause diseases. This review explores the functions of heme at the host–pathogen interface. We discuss the multifaceted roles of heme in bacterial pathogenesis and the potential for heme-targeting antimicrobial therapies. Beyond serving as a source of iron in the host environment, where iron bioavailability is limited, heme contributes to the structural stability and enzymatic functions of hemoproteins. We examine the regulatory mechanisms governing bacterial heme homeostasis in the host environment including sensing, detoxification, acquisition, utilization, and degradation pathways. Understanding how heme influences bacterial survival and virulence can lead to the development of novel therapeutic strategies that target the various essential and conserved mechanisms of heme homeostasis in bacterial pathogens. Given the rising challenge of antibiotic resistance, heme-based therapeutic interventions are promising strategies for the treatment of bacterial infections.

## Graphical abstract

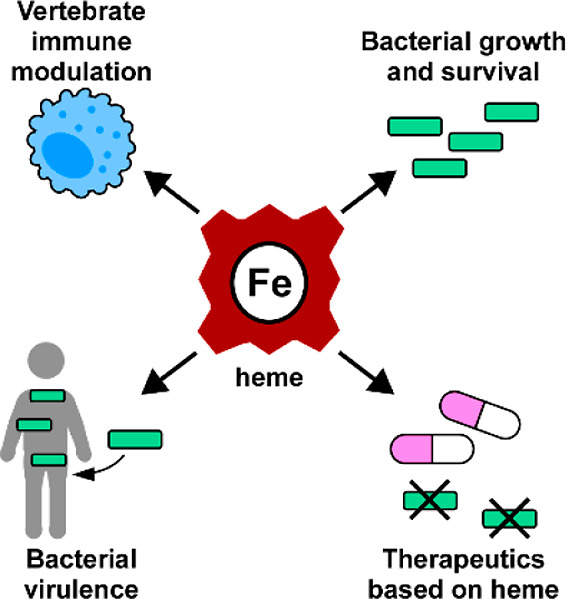

## 1 Introduction

Heme, an iron-bound protoporphyrin molecule, plays a critical role in numerous biological processes in organisms across all Kingdoms of life. Because this molecule has an iron core with high redox potential and a hydrophobic ring structure, heme commonly serves as a cofactor or redox sink for enzymes participating in oxygen transport,^1,2^ energy production,^3–5^ reactive species detoxification,^6^ and cellular signaling.^7^ During bacterial infections, heme metabolism is intricately linked to various physiological and pathological processes for both the pathogen and the vertebrate host. Due to the propensity of free iron to spontaneously oxidize and generate reactive oxygen species,^8,9^ vertebrates store 70% of their available iron as heme or in heme-bound proteins.^10^ Heme-requiring bacterial pathogens generally require 0.1–10 μM of heme for optimal growth.^11^ In humans, the concentration of labile iron in serum and interstitial fluids is roughly 10–30 μM.^12^ Moreover, the vertebrate host produces immune proteins, such as lactoferrin and calprotectin, that sequester essential trace metals away from invading pathogens, in a process known as nutritional immunity.^13^ This causes the concentration of bioavailable iron in the host environment to be even lower. Collectively, these drive pathogens to preferentially acquire heme as an iron source to satisfy their iron requirement during infection of the vertebrate host.^14–16^ Heme-requiring bacterial pathogens have, therefore, evolved complex heme acquisition, utilization, and detoxification systems to ensure proper maintenance of homeostasis. These pathways are well-conserved across many bacterial pathogens, establishing them as potential targets for antimicrobial therapies.

In this review, we outline the role of heme in bacterial pathogenesis as well as antibacterial therapies that are based on heme. The review begins by examining how heme shapes the mammalian host immune system, followed by its functions in bacterial hemoproteins including redox control, protein stabilization, and enzymatic activity. We explore diverse ways in which bacteria acquire and degrade heme and summarize advancements in the development of heme-based antimicrobials, discussing associated concerns and challenges. Finally, we provide concluding remarks on the outlook of this research field.

## 2 Mammalian Heme Metabolism

Many pathogens secrete toxins, including hemolysins, that lyse host erythrocytes to gain access to heme.^17–23^ During hemolysis, mammalian host scavenges released free hemoglobin using haptoglobin, or free heme using albumin or hemopexin.^2,24^ These heme-scavenging complexes then traverse to the spleen, where macrophages containing heme oxygenase 1 (HO-1) degrade heme to recycle iron and tetrapyrrole.^25–27^ Vertebrate hosts harbor two isoforms of heme oxygenases, HMOX1 and HMOX2.^28^ While HMOX2 is ubiquitously and constitutively expressed in most cell types,^28^ HMOX1 abundance increases in the presence of oxidative stress and serum hemoglobin levels.^29^ This positive feedback mechanism is utilized by vertebrate hosts to mitigate oxidative damage to the endothelium and tissue.^8,30–32^ HO-1 degrades heme to the linear tetrapyrrole biliverdin IX alpha (BVIXα), carbon monoxide (CO), and free iron.^33,34^ Liberated iron is then rapidly sequestered by ferritin or transported by transferrin to avoid Fenton-associated toxicity.^35,36^ In mammalian hosts, BVIXα is then reduced to unconjugated bilirubin by biliverdin reductase,^37^ which is then transported to the liver (Figure 1). Once in the liver, bilirubin can be conjugated to glucuronic acid to form bilirubin diglucuronide.^38^ Next, bilirubin diglucuronide is secreted in the gut and is converted to urobilinogen by gastrointestinal microbes such as *Clostridium spp.* ^39–41^ and *Bacteroides fragilis.* ^40^ The conversion is accomplished via bilirubin reductases. Urobilinogen is released along with bile.^42,43^ Urobilinogen can be reduced to stercobilinogen.^44,45^ Finally, the tetrapyrroles are excreted as urobilin through urine or stercobilin through feces.^45^

**Figure 1:**
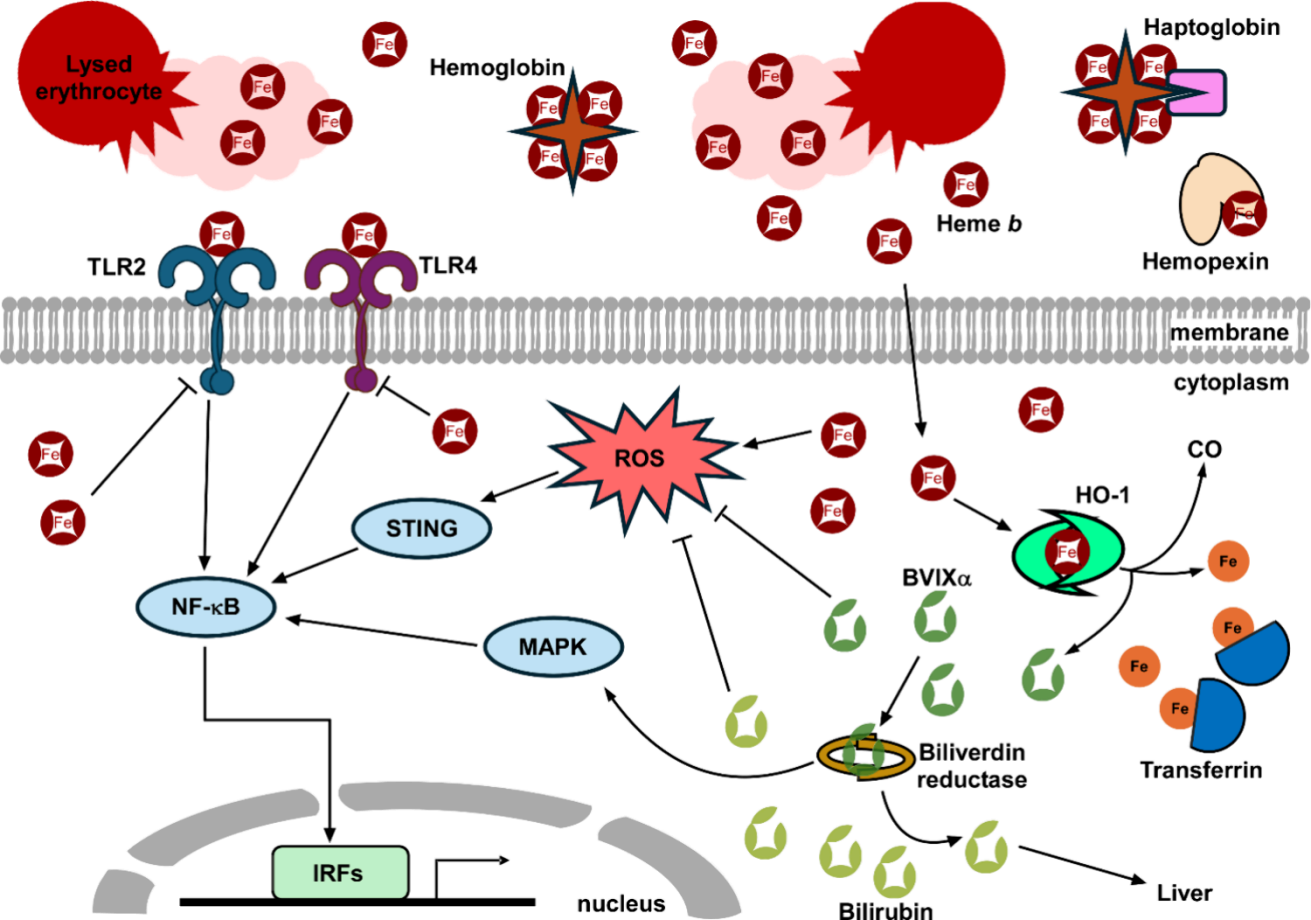
**Host heme metabolism and activation of the innate immune response.** Upon hemolysis, host erythrocytes release heme and hemoglobin, which can readily generate reactive oxygen species. Host proteins hemopexin and haptoglobin scavenge labile heme and hemoglobin for recycling and keeping them away from invading pathogens. Heme is recognized by Toll-like receptors (TLRs) 2 and 4 as a damage associated molecular pattern, which activates the NF-κB pathway to induce the production of interferon regulatory factors (IRFs). Heme also downregulates the production of TLR2 and TLR4. The reactive oxygen species generated by the cytoplasmic heme activate STING, which also activates NF-κB downstream. To limit oxidative damage, host heme oxygenases degrade heme to biliverdin IXα (BVIXα), iron, and carbon monoxide (CO). Transferrin binds to free iron. Biliverdin is then conjugated to bilirubin by biliverdin reductase, which is shuttled to the liver for downstream metabolism. Biliverdin and bilirubin are both antioxidants.

### 2.1 Heme at the Host–Pathogen Interface

To limit nutrient availability to invading pathogens within the vertebrate environment, serum hemopexin and haptoglobin play important roles to sequester bioavailable heme-iron in mammalian hosts.^46^ In vertebrates, heme serves as an immune activation signal: free, circulating heme is a damage-associated molecular pattern (DAMP) that is recognized by Toll-like receptor (TLR) 2 ^42,47,48^ and TLR4,^49–52^ triggering expression of downstream immune-responsive genes. The combination of treatment with heme and pathogen-associated molecular patterns, such as polyinosinic-polycytidylic acid (poly I:C) or bacterial lipopolysaccharides (LPS), induces inflammatory cell death in mouse bone marrow derived macrophages.^53^ Because of its oxidative property, free heme is also a proinflammatory molecule, which polarizes immune cells toward inflammatory states.^33,54,55^ To reduce the toxic effects of free heme, host HO-1 must degrade heme. Mice without HO-1 are more susceptible to bacterial sepsis when given exogenous heme before infection.^56^ However, the release of iron by HO-1 can benefit intracellular pathogens, such as *Mycobacterium tuberculosis* and *Salmonella enterica*, by increasing iron availability.^57–59^ Studies show that mice treated with HO-1 inhibitors have decreased intra-macrophage *M. tuberculosis* burden and less pneumonic lesions, but increased serum interferon gamma (IFNγ) and lower transforming growth factor beta (TGF-β) levels.^58^

In addition to heme, vertebrate heme catabolites have also been shown to have immune-modulatory effects. BVIXα is a potent scavenger of reactive oxygen and nitrogen species (ROS, RNS) within the cells, which reduces oxidative damage.^60,61^ CO has known roles in wound healing,^62,63^ vasodilation,^64,65^ platelet aggregation inhibition,^66,67^ and inhibition of pro-inflammatory cytokine production.^68,69^ Downstream of heme degradation, upon binding to BVIXα, biliverdin reductase can signal to MAPK^70^ and through PI3K/Akt to activate the NF-κB pathway,^71^ which leads to the production of proinflammatory cytokines to initiate an innate immune response cascade.^50,72^ This has been postulated to be a reason that BVIXα is so readily and rapidly converted to bilirubin in mammals. Despite this evidence, other studies have found that biliverdin reductase plays multiple roles in reducing the expression of TLR4 on murine macrophages.^50,54^ Bilirubin serves as an antioxidant in low concentrations (7 ng/mg of protein), but a pro-oxidant in high concentrations (25 ng/mg protein),^73–76^ which causes ROS-mediated neurotoxicity, particularly in infants. Conjugated bilirubin is an agonist of peroxisome proliferator-activated receptor alpha (PPARα),^77,78^ which is a known receptor in the gastrointestinal tract to drive immune response in vertebrates.^79,80^ Taken together, these findings underscore how heme and its degradation catabolites play various roles in shaping vertebrate immune responses.

## 3 Bacterial Hemoproteins

Beyond serving as the major source of iron in the vertebrate host environment, heme is crucial for structural stability and therefore the functions of essential bacterial hemoproteins, which promote bacterial survival and virulence. Heme-requiring bacterial infections are intricately linked to their abilities to utilize and detoxify heme.^81,82^

### 3.1 Cytochromes: Energy Generation and Structural Stability

Cytochromes are hemoproteins with multifaceted functions, including electron transfer, detoxification of reactive species, and generation of the membrane potential, which rely on the redox cycling abilities of their heme ligand between the Fe^2+^ and Fe^3+^ states.^83,84^ Cytochromes are classified by their heme prosthetic groups; for example, cytochrome *a* contains the modified heme moiety, heme *a*. Cytochrome *c* maturation is contingent upon the binding of heme *b* to a conserved heme-binding CXXCH motif, forming heme *c,* ^85^ which is facilitated by the cytochrome *c* maturation system to deliver heme *b* to nascent cytochromes before its insertion into the membrane.^86–88^ In contrast, cytochromes *b*, the major components of the respiratory chain, contain two axial histidine residues to bind heme *b*. Aerobic bacterial pathogens, similar to eukaryotic organisms, possess cytochrome oxidases in their cell membranes that function in cellular respiration.^89^ An inability of apo-cytochromes to effectively participate in menaquinol oxidation and catalysis of O_2_ into H_2_O forces facultative anaerobes to instead generate energy through fermentation.^90^ In *Staphylococcus aureus*, a strain that lacks cytochrome *bd* (Δ*cydB*) shows reduced growth *in vitro* and reduced colonization in the heart and liver of a systemic mouse infection model.^91^ Colonization of *S. aureus* strains deficient in heme biosynthesis shows reduced bacterial burden in an organ-specific manner,^92^ supporting the role of heme in proper cytochrome functioning. Additionally, a *Salmonella enterica* strain lacking *cydAB* and *cyoABCD*, encoding for cytochrome *bd* and *bo* quinol oxidase complexes, respectively, has a significant growth defect *in vitro* compared to wildtype strain, and the Δ*cydAB* strain is significantly attenuated in murine small intestines and spleens.^93^

Beyond contributing to enzymatic function, the porphyrin ring of heme provides structural integrity to restrain prosthetic groups of cytochromes, independent of the iron atom.^94^ Mutation of heme-iron binding residues in cytochrome *c* shows that the overall protein stability is marginally enhanced.^95^ However, the secondary and tertiary structures are partially lost.^95^ Work done on hemoproteins that contain heme *b*, which include cytochrome P450 family proteins, shows that the stability of holo-hemoproteins is positively correlated with their affinity for the heme ligands.^96^ In *Escherichia coli*, mutations in interacting residues Methione-393 and Arginine-391 of axial heme ligand *b*_558_ of cytochrome *bd* demonstrate reduced midpoint electrochemical potential, leading to decreased redox cycling *in vitro.* ^97^ This showcases the contribution of heme and interacting residues to proper hemoprotein structures and activities.

Bacterial cytochrome oxidases serve dual functions in detoxifying environmental assaults. These stressors include byproducts generated through the electron transport chain, heme catabolism, and anaerobic respiration.^98,99^ *E. coli* cytochrome *bd*-type oxidases, which contain two heme *b* and a heme *d* molecule, have a stronger affinity for oxygen compared to cytochrome *bo*-type oxidases, which contains heme *b* and heme *o.* ^89^ This allows facultative anaerobes to adjust to microaerobic or hypoxic environments. *E. coli* cytochrome *bd*-II, which relies on heme *d*_1_ to enzymatically convert H_2_O_2_ to O_2,_ ^100^ is reminiscent of the periplasmic cytochrome *c* peroxidases, which bind two molecules of heme *c*. Early reports found that cytochrome *bo*_3_ in *E. coli* may be the main oxidase in the presence of CO,^101,102^ but it was since discovered that cytochrome *bd*-I is the most resistant to CO-induced inhibition of heme *d.* ^103,104^ Corroborating that finding, studies in *E. coli* and *S. enterica* demonstrate that cytochrome *bd* plays a role in promoting bacterial growth under nitrosative stress.^105–108^ Nitric oxide (NO) binds heme with higher affinity than O_2_ to heme,^109^ which inhibits cytochromes responsible for aerobic respiration.^110^ Cytochrome *bd* in *E. coli* and *S. enterica* exhibits a higher NO dissociation rate than cytochrome *bo,* ^111–113^ facilitating rapid recovery of bacterial respiration in the presence of NO and emphasizing the crucial role of heme in electron transport and bacterial survival under stress conditions.

### 3.2 Detoxification Using the Redox Potential of Heme

Heme-containing cytochrome *c* peroxidases and catalases are important for bacteria that possess them to detoxify ROS endogenously produced during aerobic respiration and from environmental sources, such as the host immune response.^114^ Expression of heme-catalases may be regulated by H_2_O_2_ or O_2_-sensing transcriptional regulators. Pathogenic bacteria lacking catalase have attenuated pathogenesis, indicating the importance of hemoproteins in promoting bacterial survival. *S. aureus* strains lacking catalase are unable to survive oxidative stress within mouse peritoneal macrophages.^115^ In conjunction, the intracellular *S. aureus* catalase level positively correlates to host production of inflammatory cytokines.^116^ Catalase is required for virulence in *Leptospira interrogans*, as strains lacking *katE* are attenuated in a hamster acute leptospirosis model.^117^ *Campylobacter jejuni* without the heme chaperone Cj1386 to deliver heme to catalase has diminished catalase activity and demonstrates reduced colonization in broiler chick ceca and neonatal piglet gastrointestinal tracts.^118^ These examples illustrate that heme-catalase is important for bacterial proliferation and the onset of an infection.

Along with cytochrome *bd*, flavohemoglobins catalyze the degradation of NO to detoxify it. Flavohemoglobin Hmp is required for *S. aureus* biofilm formation under nitrosative stress.^119^ In *E. coli* grown in the presence of lethal concentrations of sodium nitroprusside, strains with heterologous expression of the heme-binding domain in Hmp on a multicopy plasmid confers growth advantage over strains that lack Hmp or strains that heterologously express the flavin-binding domain instead.^120^ However, full resistance to NO inhibition *in vitro* requires holo-Hmp.^120^ Production of *S. enterica* serovar Typhimurium Hmp is increased in the presence of NO, and contributes to the intracellular virulence in mouse infection models and in human macrophages.^121,122^ An Hmp-null *S. enterica* serovar Typhimurium strain is auxotrophic for cysteine and branched chain amino acids,^123^ highlighting the role of Hmp in detoxifying NO stress under host environments that could be scarce for bioavailable amino acids. A unique, two-domain flavohemoglobin in *M. tuberculosis* is involved in detoxifying metabolic byproduct d-lactate, which is generated during lipid peroxidation under aerobic respiration.^124^ Under the accumulation of d-lactate, *M. tuberculosis* flavohemoglobin uses d-lactate as a reductant, instead of NADPH, and transfers electrons through FAD to heme, thereby decreasing cellular oxidative stress.^124,125^ Collectively, these findings emphasize the vital role of heme-containing proteins in enhancing bacterial survival against environmental assaults, making them potential targets for therapeutic interventions.

### 3.3 Heme Sensors: Ligand-Induced Conformational Change

Heme is toxic at elevated concentrations because it generates reactive species in the presence of redox-sensitive compounds,^8,24,126^ oxidizes lipids and lipoproteins,^10,24^ and disrupts membrane function by incorporating into the membrane.^10,24,127^ This emphasizes the necessity for heme detoxification systems in bacterial pathogens to maintain homeostasis while exposed to high concentrations of heme during infection of the host.^10,11^ Many bacteria maintain heme-responsive transcriptional regulators that use heme as a direct ligand to differentially express genes participating in heme detoxification. In these transcriptional regulators, heme binding induces conformational change to achieve protein function, leading to the modulation of heme efflux pump expression.

#### 3.3.1 Hrt

The heme-response element HssRS in *S. aureus* and *Bacillus anthracis* is a two-component system. The transmembrane heme-sensing HssS histidine kinase dimerizes upon recognition of heme and autophosphorylates and phosphorelays to the cytoplasmic response regulator HssR (Figure 2A). Phospho-HssR then binds to the promoter region of *hrtAB* and activates its transcription.^128,129^ HrtB is an ATP-synthase binding cassette (ABC) membrane permease and HrtA is the associated ATPase, which together form a functional heterodimer of homodimers.^130^ Dimerization of HrtB forms a heme-binding site,^131^ and the glutamate residues at the binding site recognize the heme ligand.^131^ HrtA hydrolyzes ATP to change the conformation of the HrtB heme-binding site to facilitate substrate efflux across the membrane.^131^ *hssRS* is encoded in the opposite direction of the *hrtAB* locus and HssR positively regulates *hrtAB* mRNA abundance.^91,128,131,132^ HssS recognizes non-iron metallo-protoporphyrin IX (PPIX), but not labile metal ions or PPIX alone.^91^ It has recently been reported that HssS senses heme within the membrane, instead of extra- or intracellular heme,^133^ as the heme binding pocket of HssS is located between the extracellular space and the membrane. Deletion of *hrtAB* in *S. aureus* leads to increased secretion of immunomodulatory factors and hypervirulence in the liver.^91,134^

**Figure 2:**
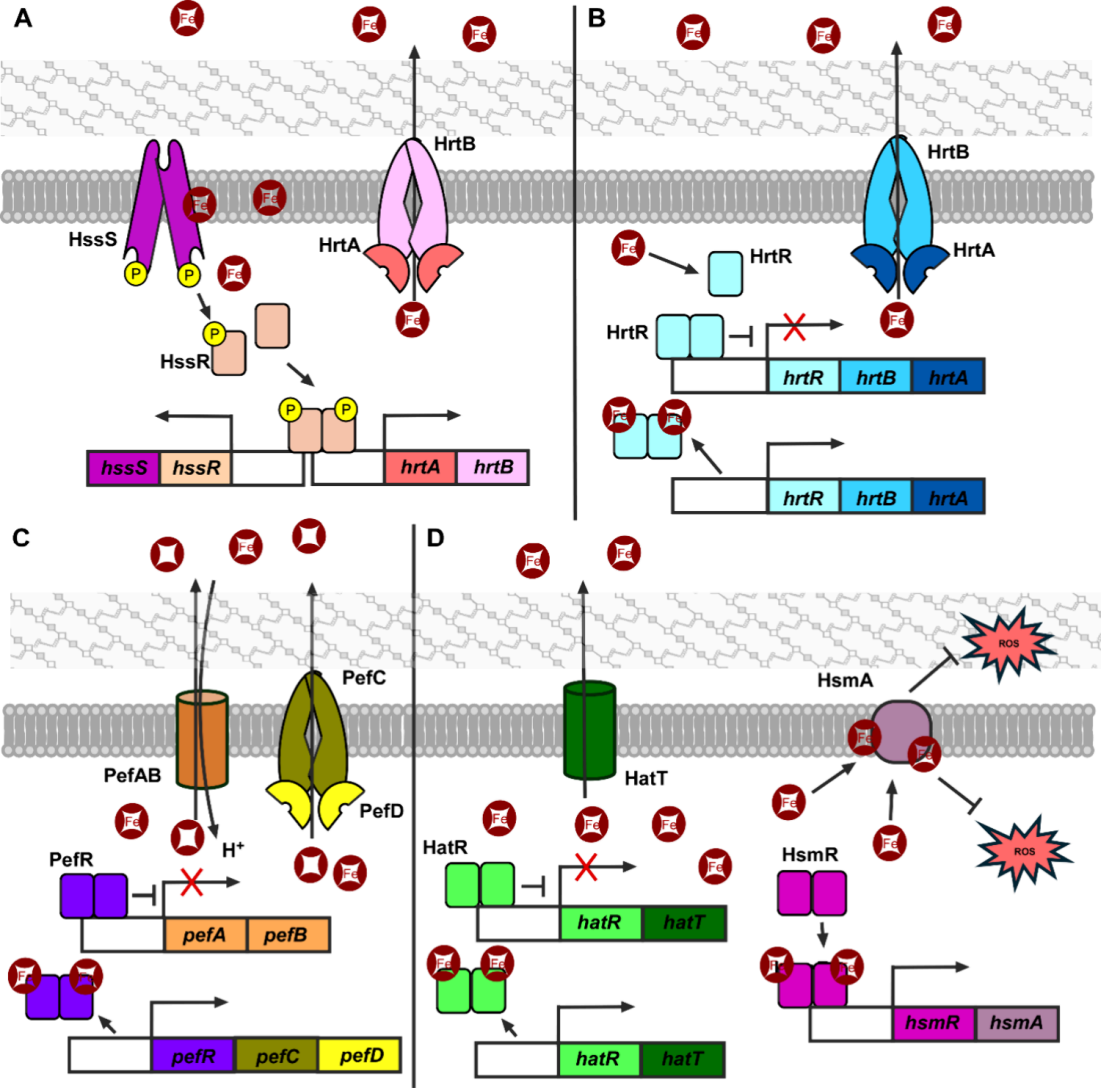
**Heme sensing and detoxification systems.** (A) *Staphylococcus aureus* heme-sensing two-component element HssS, upon binding heme, phosphorelays to HssR. Phosphorylated HssR dimerizes and translocates to the promoter regions of the *hrtAB* and *hssRS* operons. ABC membrane permease complex HrtAB then effluxes excess heme out of the cytoplasm. (B) Apo *Lactococcus lactis* HrtR binds to the promoter region of the *hrtRBA* operon to repress expression. Heme binding induces a conformational change to allow holo-HrtR to dissociate from the *hrtRBA* promoter. (C) *Streptococcus agalactiae* MarR-type heme-sensing regulator PefR derepresses the *pefAB* and *pefRCD* operons when it binds heme. PefCD is an ABC membrane PPIX efflux pump. PefAB is a MFS transporter. (D) *Clostridioides difficile* heme-sensing regulator HatR derepresses *hatRT* when bound to heme. HatT is an MFS transporter. HsmR binds heme to activate expression of *hsmRA.* HsmA is a membrane-bound heme storage and defends against oxidative stress.

In *Lactococcus lactis*, transcriptional activation of *hrtBA* is instead achieved by derepression. A cytoplasmic TetR family transcriptional regulator HrtR negatively regulates transcription of *hrtBA* (Figure 2B). Expression of the *hrtRBA* operon is highly induced upon bacterial exposure to heme.^135–137^ HrtR possesses nine conserved helices^136^ and functions as a homodimer independent of heme binding.^136^ Each protomer binds two major grooves of DNA via helix-turn-helix motifs^136^ (Figure 3A). Upon heme docking into the HrtR hydrophobic pocket, interaction with two histidine residues induces a coil-to-helix transition.^138^ The conformational change in the heme binding α helix 4 creates steric hindrance in the positioning of helix 3 and pushes the α helix 3 at an angle^138^ (Figure 3B). This pushes the centers of the DNA recognition domain and the residues on the major groove away from each other, thus allowing HrtR to dissociate from the 15-bp promoter region of *hrtRBA.* ^138^ In *Enterococcus faecalis*, expression of *hrtAB* homologues is also mediated by a TetR regulator, FhtR, but FhtR does not share significant sequence homology with *L. lactis* HrtR.^139^ In addition to free heme and hemoglobin in host blood, *E. faecalis* FhtR responds to heme biosynthesized by *E. coli* ^139^ and host heme liberated by *Clostridioides difficile.* ^140^ This illustrates the flexibility of bacterial heme sensors responding to environmental cues from diverse sources.

**Figure 3:**
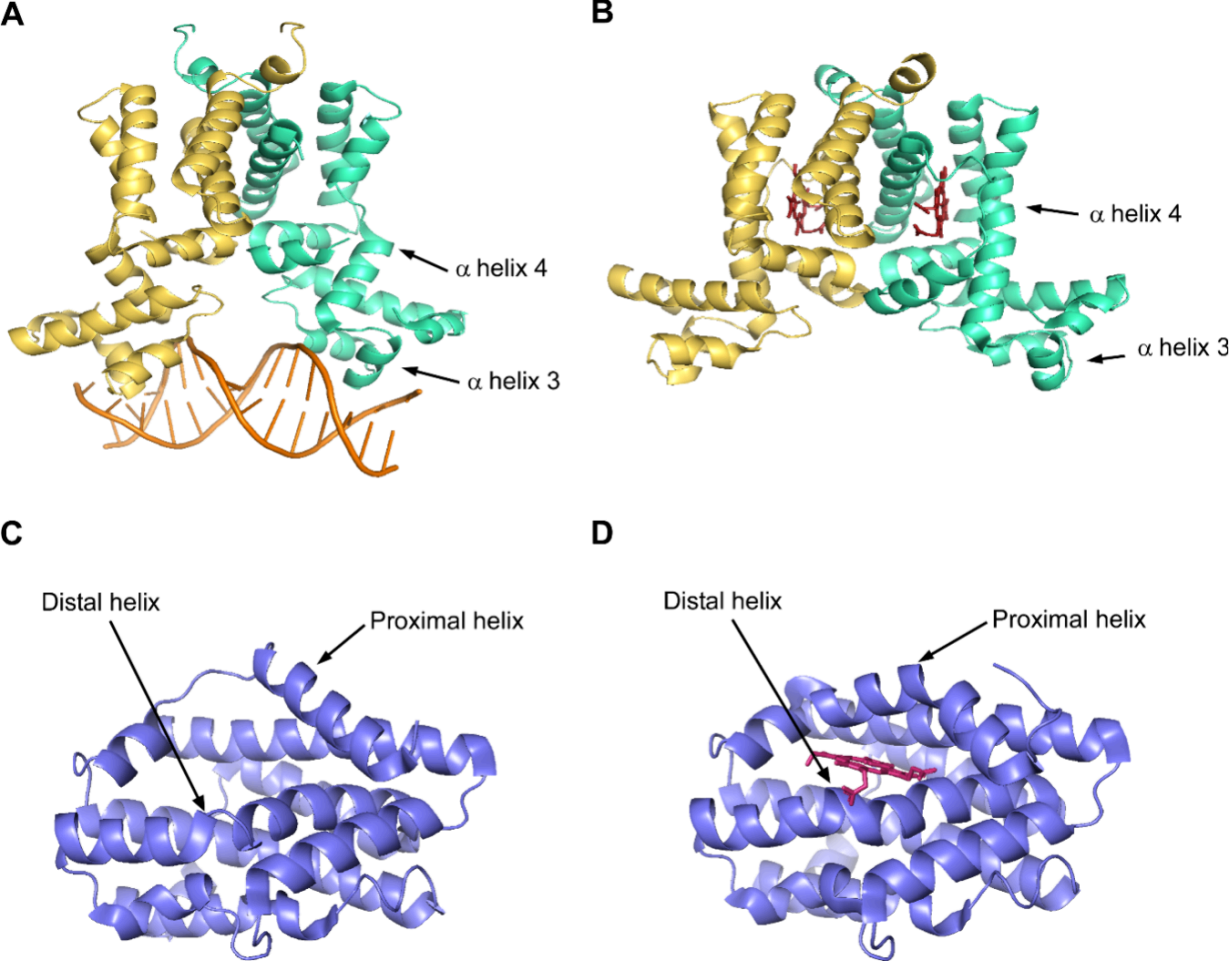
**Structural rearrangement of hemoproteins upon ligand binding.** Crystal structures of *Lactococcus lactis* HrtR and *Corynebacterium diphtheriae* HmuO. (A) The helix–coil–helix structure of α helix 4 on each of the monomers allows apo-HrtR to bind to the target DNA (PDB: 3VOX). (B) Holo-HrtR dimer. Binding of heme ligand in the hydrophobic pocket induces a coil-to-helix conformation change of α helix 4, which pushes the α helix 3 outward, leading to distancing between the DNA-binding residues and the DNA major groove (PDB: 3VP5). (C) Apo-HmuO proximal and distal helices to the heme-binding pocket are in helix–coil–helix conformations (PDB: 4GOH). (D) Holo-HmuO distal and proximal helices take on the coil-to-helix change and form a closed heme pocket (PDB: 1IW1). Red, heme. Yellow and green are HrtR monomers. Orange, *hrtRBA* promoter DNA. Blue, HmuO. (A) and (B) are adapted from *The Journal of Biological Chemistry*, *287* (36), Sawai, H. et al. Structural basis for the transcriptional regulation of heme homeostasis in *Lactococcus lactis*, 30755–30768, Copyright Elsevier (2012).^136^ (C) is adapted from *The Journal of Biological Chemistry*, *288* (48), Unno, M. et al. Structures of the substrate-free and product-bound forms of HmuO, a heme oxygenase from *Corynebacterium diphtheriae*: X-ray crystallography and molecular dynamics investigation, 34443–34458, Copyright Elsevier (2013).^255^ (D) is adapted from *The Journal of Biological Chemistry*, *279* (12), Hirotsu S. et al. The crystal structures of the ferric and ferrous forms of the heme complex of HmuO, a heme oxygenase of *Corynebacterium diphtheriae*, 11937–11947, Copyright Elsevier (2004).^256^ All distributed under CC-BY license 4.0 (https://creativecommons.org/licenses/by/4.0/). This figure is produced in PyMOL.

#### 3.3.2 PefR

In the opportunistic pathogen *Streptococcus agalactiae*, the Pef regulon comprises multidrug resistance efflux pumps regulated by PefR, a Multiple Antibiotic Resistance Regulator (MarR)-type heme-responsive regulator (Figure 2C). PefR regulates the operons *pefAB* and *pefRCD*, which encode for the heme and PPIX efflux pumps.^141^ Apo-PefR binds to its target DNA sequence via a winged helix-turn-helix motif that consists of two helices and two strands, as opposed to the all-helix motif in HrtR.^142^ A stable complex with the DNA is formed as the helices bind the major grooves and the strands insert into the minor grooves.^142^ Like the mechanism elucidated for HrtR, holo-PefR undergoes a conformational change upon heme ligand binding. The heme ligand provides a steric force pushing the recognition domain further apart, causing PefR to dissociate from the target DNA, resulting in derepression of *pefAB* and *pefRCD.* ^143^ Interestingly, ferrous heme-PefR can bind CO, a byproduct of heme degradation.^143^ This is the first report of a heme-sensing response protein performing similarly to an environmental-sensing hemoprotein, laying the groundwork for further studies of multifunctional heme-binding proteins. Although the biological relevance of this interaction remains elusive, this could point to a feedback mechanism between heme catabolism and heme sensing.

#### 3.3.3 HatR and HsmR

The enteric pathogen *C. difficile* possesses a TetR family heme transporter-regulator pair that operates differently from the HrtR system. *C. difficile hatR* is in the same operon as a singular heme-responsive major facilitator superfamily transporter HatT, similar to the HrtAB system^144^ (Figure 2D). Although *C. difficile* lacks the genes encoding for heme biosynthesis, the bacterium experiences heme stress during gastrointestinal tract colonization.^144^ Blood hemoglobin levels positively correlate with the onset of *C. difficile* infection.^144^ Expression of the *hatRT* operon is derepressed in the presence of heme. Although no crystal structure of HatRT has been reported to date, HatT is predicted to be a passive uniporter that transports its ligand down the concentration gradient without energy consumption.^145^ Not only do *hatR* and *hrtR* share low sequence homology, *C. difficile* HatR recognizes heme with only one histidine residue, and HatR recognition is specific to heme, not to PPIX.^144^ Strains lacking *hatRT* have decreased colonization in the murine infection model.^144^

In addition to the HatRT system, *C. difficile* encodes for another MarR-type transcriptional regulator, HsmR (Figure 2D), that activates the expression of the operon *hsmRA* upon sensing heme.^146^ *hsmR* sequence suggests that HsmR harbors conserved structures of canonical MarR family proteins. Uniquely, cytoplasmic heme-binding HsmR induces HsmA, a membrane-bound heme storage protein. Instead of pumping out excess heme, HsmRA sequesters heme to the membrane, presumably using redox cycling of heme to defend against redox damage generated by the immune system or exposure to antibiotics.^146^ The mechanism of ligand-induced conformational changes of these heme sensing and detoxification response elements underlines the specificity of bacterial transcriptional responses. Although the mechanisms employed by each system may differ, the fact that bacterial pathogens found across diverse infection niches have independently evolved heme sensor-detoxification systems emphasizes the essentiality of maintaining heme homeostasis during an infection.

## 4 Bacterial Heme Acquisition

There are two main modes to acquire heme: endogenous biosynthesis and import from exogenous sources. While many bacterial pathogens can endogenously synthesize heme to satisfy the biochemical need to power hemoproteins in a heme-depleted environment, production of the enzymes required in the multistep heme biosynthesis process makes it energetically costly. Heme from endogenous or exogenous sources can be transported to populate hemoproteins, such as cytochromes and catalases, as previously described in Section 3. In bacteria that possess heme degradation enzymes, heme can also be degraded to serve as an iron source. Pathogens that are dependent on heme availability to proliferate and colonize the host have evolved to have diverse, specialized heme acquisition systems to hijack heme from the host or to cross-feed off heme-producing bacteria that occupy the same niche.

### 4.1 Bacterial Heme Biosynthesis

The divergent bacterial heme biosynthesis pathways have been extensively reviewed previously.^147–152^ Eukaryotic and prokaryotic heme syntheses share a common committed precursor, 5-aminolevulinate acid (ALA). Most prokaryotes, with the notable exception of alphaproteobacteria, generate ALA through the 5-carbon (C5) route, where glutamyl-tRNA is produced from carbon cycling via the citric acid cycle, and glutamyl-tRNA reductase (GtrR) and glutamate-1-semialdehyde-2,1-aminomutase (GsaM) sequentially convert glutamyl-tRNA into ALA. GtrR catalyzes the rate-limiting first step of bacterial heme biosynthesis,^151,153–157^ and its abundance is post-transcriptionally regulated by the presence of heme.^153,154,157–161^ In *Corynebacterium diphtheriae*, expression of GtrR is inhibited when the heme-sensing two-component system ChrSA recognizes hemoglobin.^162^ In *S. aureus* and *Bacillus subtilis*, GtrR abundance is also repressed by membrane-bound protein HemX.^159,163^ *S. aureus* GtrR is further regulated by serine/threonine kinases,^153^ which controls cell growth cycles.^164–166^

Three conserved enzymes convert eight molecules of ALA to uroporphyrinogen III (uro’gen).^150,167^ After generation of uro’gen, the pyrrole schematics branch off. The siroheme pathway is utilized to produce other tetrapyrroles, such as cobalamin or heme *d,* ^168–171^ a modified moiety of heme *b.* ^172^ Archaea and selective sulfate-reducing bacteria also utilize the siroheme pathway for heme *b* production, which is proposed to evolve from anaerobic heme biosynthesis.^149,173^ In nonsiroheme pathways, uro’gen is converted into coproporphyrinogen III (copro’gen) by uroporphyrinogen decarboxylase, where the biosynthesis once again branches into the classical protoporphyrin-dependent (PPD) or the recently described coproporphyrin-dependent (CPD) pathway.^151^ The PPD pathway is used by eukaryotes and Gram-negative organisms; Gram-positive organisms use the CPD pathway,^174^ except for *Nitrospira defluvii.* ^175,176^ Interestingly, although the heme auxotroph *Bacteroides spp.* do not synthesize their own heme, they harbor a conserved *uroS* within their genomes. *B. fragilis* uroporphyrinogen III synthase (UroS) allows the bacterium to grow on heme or PPIX *in vitro*, and contributes to its survival in mouse intra-abdominal infection and gut colonization models.^177^

In the PPD pathway, copro’gen is converted to protoporphyrinogen IX by coproporphyrinogen decarboxylase (CgdC) aerobically or coproporphyrinogen dehydrogenase (CgdH) in facultative and obligate anaerobes.^150,173^ This dual-decarboxylation step generates two molecules of carbon dioxide. Protoporphyrinogen IX is conjugated as PPIX by protoporphyrinogen oxidase.^178^ Heme biosynthesis can be regulated at the PPIX level. Several PPIX-specific efflux pumps have been identified in Gram-negative bacteria,^179^ including PefCD as mentioned above. In the final step of PPD heme *b* production, an iron atom is inserted into PPIX by protoporphyrin ferrochelatase (PpfC). However, PpfC does not discriminate against non-iron divalent metal for insertion,^152,180^ potentiating this enzyme as a drug target.

On the contrary, *Firmicutes* and *Actinobacteria* do not accumulate protoporphyrin as intermediates during heme production, but instead accumulate coproporphyrin.^181–184^ In the CPD pathway, copro’gen is conjugated into coproporphyrin III by coproporphyrinogen oxidase aerobically, and hydrogen peroxide is released as a byproduct. In the penultimate step of heme *b* production, the CPD ferrochelatase CpfC converts coproporphyrin III to coproheme III, which has two carboxyl groups on the A and B pyrrole propionates compared to those of heme *b*. Finally, coproheme III is decarboxylated by aerobic coproheme decarboxylase ChdC or anaerobic heme synthase AhbD.^149,185^

Efforts to identify a designated oxygen-independent coproporphyrinogen oxidase have resulted in reports of two distinct, yet similar, FAD-dependent enzymes. CgoX in *S. aureus* ^186^ and CgoN in *Bacillaceae* ^187^ are solely responsible for both aerobic and anaerobic catalysis of this step in their respective organisms. CgoN is almost exclusively found in *Bacillaceae,* ^187^ however, *S. aureus* also encodes a CgoN homologue that does not participate in heme biosynthesis.^186^ Further investigations into how these paralogues have evolved and how genetic redundancy occurs are warranted and may shed light on key mechanisms driving the evolution of bacterial heme biosynthesis.

### 4.2 Bacterial Heme Uptake

There are several detailed and comprehensive reviews on Gram-positive and Gram-negative bacterial heme uptake systems.^148,188,189^ Heme uptake systems can be roughly categorized into direct binding to heme receptors and hemophore-mediated uptake. Here, selective models of each category are briefly discussed.

#### 4.2.1 Direct Binding and Relay of Heme: *S. aureus* Isd System

*S. aureus* secretes leukocidins in part to gain access to intracellular host hemoglobin, heme, and the haptoglobin–hemoglobin complex.^17^ Heme is bound and transported across the membrane via the iron surface determinant (Isd) system,^190,191^ which is comprised of three main fractions: the cell wall-anchored receptors, cell membrane-embedded transporters, and cytoplasmic heme oxygenases (Figure 4A). IsdA, IsdB, and IsdH are peptidoglycan-anchored receptors which protrude into the extracellular space.^190,192^ Anchoring of these surface receptors is mediated by cysteine transpeptidase sortase A.^191^ The ligand binding specificity is determined by their NEAr-iron Transporter (NEAT) domains, which consist of conserved nine β-sandwich fold motifs harbored by many putative ferric iron siderophore transporter genes.^193^ IsdA contains one NEAT domain, IsdB two,^194^ and IsdH three.^195^ The tyrosine residue within the hydrophobic NEAT domain facilitates heme-iron binding.^196^ Heme binding to these receptors is coordinated by the C-terminal NEAT domain, while the N-terminal domains function to recognize heme-protein complexes.^193,197^ IsdA directly sequesters free heme,^198^ IsdB preferentially binds to hemoglobin,^194,199–201^ and IsdH binds to haptoglobin-bound hemoglobin.^202^ Differential binding preferences of these surface receptors allow *S. aureus* to utilize diverse sources of heme. The captured heme from IsdB and IsdH is relayed unidirectionally to IsdA,^200,203^ which is then relayed to IsdC,^204^ the fourth receptor which is embedded within the peptidoglycan layer. Unlike the surface receptors, IsdC is anchored via sortase B, whose encoding gene is located within the *isdCDEFsrtBisdG* operon.^190,191,205,206^ Transcription of *isd* genes is negatively regulated by the ferric uptake regulator Fur, underscoring the homeostatic balance between heme and iron levels in bacteria. IsdC relays heme to the membrane-bound transporter complex. Heme is then passed to lipoprotein receptor IsdE,^207,208^ which then shuttles the heme to the transmembrane permease IsdF powered by ATPase FhuC.^209^ *isdD* in the same operon encodes for a transmembrane protein of unknown function which forms a complex with IsdF. Finally, IsdF transports heme into the cytoplasm where it can be inserted into hemoproteins or degraded by heme oxygenases IsdG and IsdI.^210–212^

**Figure 4:**
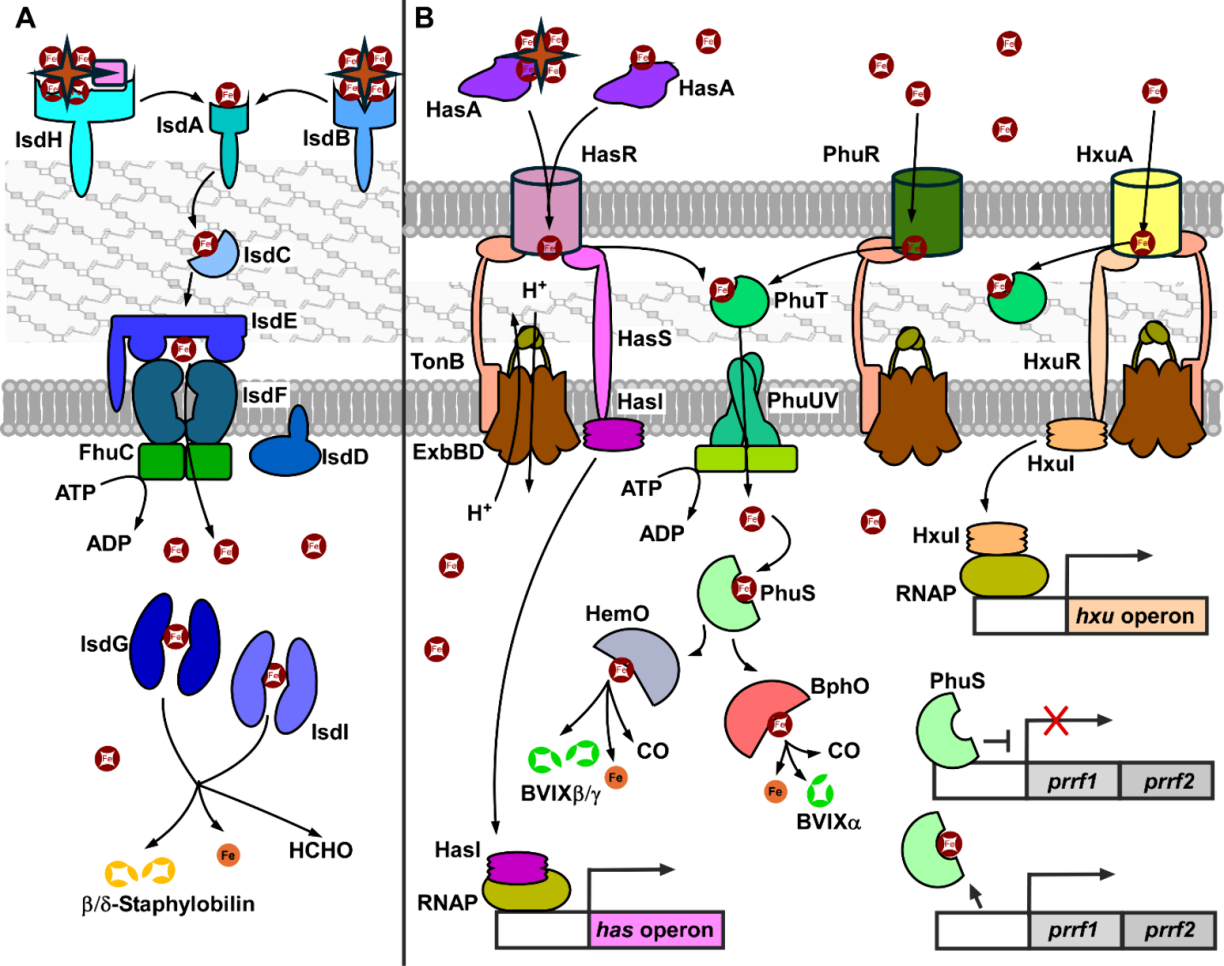
**Heme uptake systems.** Receptor-mediated uptake is represented by the iron surface determinant (Isd) system in the Gram-positive pathogen *Staphylococcus aureus*. Hemophore-mediated, TonB-mediated uptake is represented by the *Pseudomonas* heme uptake (Phu), the heme assimilation system (Has), and the hemopexin-binding Hxu system in the Gram-negative pathogen *Pseudomonas aeruginosa*. (A) *S. aureus* cell-wall-bound receptors IsdA, IsdB, and IsdH recognize heme, hemoglobin, and haptoglobin–hemoglobin complex. Heme is transferred to IsdC then through the ABC membrane permease complex IsdEFFhuC into the cytoplasm. Heme oxygenases IsdG and IsdI degrade heme to staphylobilin, iron, and formaldehyde. (B) *P. aeruginosa* HasA is a secreted hemoprotein that binds extracellular heme and hemoglobin and transfers them across the outer membrane by the TonB-ExbBD-dependent receptor HasR. HasR relays heme to periplasmic binding protein PhuT, then to the ABC permease complex PhuUV. Cytoplasmic heme is chaperoned by PhuS to heme oxygenases HemO and BphO, breaking heme to iron, carbon monoxide, and biliverdin IX. Outer membrane receptors PhuR and HxuA both transport heme to PhuT. Ligand-bound HasR inactivates the anti-σ HasS to release the σ factor HasI, inducing *has* operon expression. Heme binding of HxuA similarly triggers HxuR to release σ factor HxuI to recruit RNA polymerase to the *hxu* operon promoter. PhuS also negatively regulates *prrF* operon expression.

#### 4.2.2 Hemophore-Mediated Intake: *P. aeruginosa* Has System

There are three nonredundant heme uptake systems in *P. aeruginosa*: the hemophore-mediated Has system and the direct heme-binding Phu and Hxu systems (Figure 4B). HasA­(p) is a soluble hemophore that is secreted by the type 1 secretion system^213,214^ and is identified in many other Gram-negative organisms.^214,215^ HasA recognizes both free heme and hemoglobin-bound heme in a 1:1 ligand to protein ratio. HasA then transfers heme to HasR, the TonB-dependent receptor anchored at the outer membrane.^216^ The transfer of heme from HasA to HasR is ATP-independent,^217^ but is dependent on the conformational rearrangement of the hydrogen bonds between heme-coordinating residues histidine and tyrosine on HasA with the bis-histidine residues on HasR.^216,218,219^ Transport of substrates across the double membrane in Gram-negative organisms rely on the TonB-ExbB-ExbD complex coupled proton motive force.^220^ The transcriptional regulation of the Has system is self-activating; binding of heme to HasR induces release of extracytoplasmic function σ factor HasI upon inactivation of anti-σ factor HasS.^221^ RNA polymerase is recruited along with HasI to activate transcription of the *has* operon,^221,222^ marking a positive feedback mechanism. The Has system is the primary extracellular heme sensor, while the Phu system is the main importer.^223,224^ Since the Has system of *P. aeruginosa* does not have a periplasmic component, once heme is transported through the outer membrane, it is hypothesized that HasR then delivers heme to the Phu system.^225^

PhuR is the second outer membrane heme receptor in *P. aeruginosa*, but contains the same His-Tyr heme-coordinating motif as HasA.^226,227^ After PhuR shuttles heme past the outer membrane via TonB-mediated transport, periplasmic heme-binding PhuT accepts heme from PhuR, and presumably HasR.^225^ Heme is finally relayed to the ABC inner membrane permease complex PhuUV. Once within the cytoplasm, heme is chaperoned to power hemoproteins or to heme oxygenase HemO by PhuS.^228^ Heme chaperone PhuS homologues are reported in *Yersinia enterocolitica,* ^229^ *Shigella dysenteriae,* ^230,231^ *Yersinia pestis,* ^232,233^ and *E. coli* *,* ^234,235^ among others.

Notably, apo-PhuS binds DNA at the promoter of *prrF1* of the PrrF1 and PrrF2 tandem sRNAs.^236,237^ PrrF sRNAs modulate mRNA degradation of iron-responsive genes by complementing these sequences.^238,239^ Transcription of *prrF*s is Fur-regulated.^239^ *prrF1* and *prrF2* together allow the transcription of *prrH*, a heme-responsive sRNA which mediates mRNA expression of pyochelin biosynthesis genes.^240,241^ The DNA binding site of apo-PhuS overlaps with the Fur binding site.^236,237^ Therefore, the regulation of *prrF* sRNAs, and by extension the iron-responsive siderophore biosynthesis genes, is affected by levels of both iron and heme. This underscores the tight regulation of heme/iron homeostasis through negative feedback. Additionally, this emphasizes how hemoproteins can be multifunctional, and novel functions of many already-identified hemoproteins have yet to be defined.

The Hxu system has been described only recently. Similar to the Has system, the Hxu system primarily functions in cell surface signaling.^242^ HxuA, the outer membrane-bound, TonB-dependent HasR homologue, binds heme directly. This triggers the release of σ factor HxuI from the anti-σ HxuR, which are homologues of HasI and HasS, respectively.^242^ In the absence of HasR, HxuA production is increased,^243^ suggesting that the Hxu system compensates for the loss of the Has system to some degree. Interestingly, induction of the *hxuIRA* operon is responsive to more than heme or heme-bound proteins; *hxuI* is upregulated in the presence of the iron-sequestering transferrin in a HxuA-dependent manner.^244^ Further, *hxuI* expression is also induced under hypoxic conditions and in the presence of ROS and NO.^245^ Overexpression of *hxuI* leads to upregulation of genes involved in siderophore biosynthesis, iron uptake, denitrification, and virulence factors, among others.^245,246^ *P. aeruginosa* clinical isolates from bloodstream infections express the Hxu system at a higher level compared to wound isolates.^244^ Consistent with this, a laboratory strain expressing 35 times higher *hxuIR* and four times higher *hxuA* than a wildtype strain is more pathogenic compared to wildtype in murine bloodstream infections, while a *hxuIRA-*null strain is attenuated,^244^ and Δ*hxuI* mutant demonstrates reduced bacterial burden in mouse lung and subcutaneous infections.^245^ Taken together, this underscores the requirement for heme in bacterial fitness and pathogenesis and establishes a complex regulatory network across heme-responsive signaling systems, iron/heme homeostasis, and pathways whose effectors include hemoproteins.

## 5 Bacterial Heme Degradation

Once bacterial pathogens acquire and shuttle heme from the environment into their cytoplasm, heme can be used to populate endogenous hemoproteins, or can be degraded to be used as an iron source or to detoxify heme stress.^247^ These heme degradation enzymes are classified based on their resulting heme catabolites (Figure 5).

**Figure 5:**
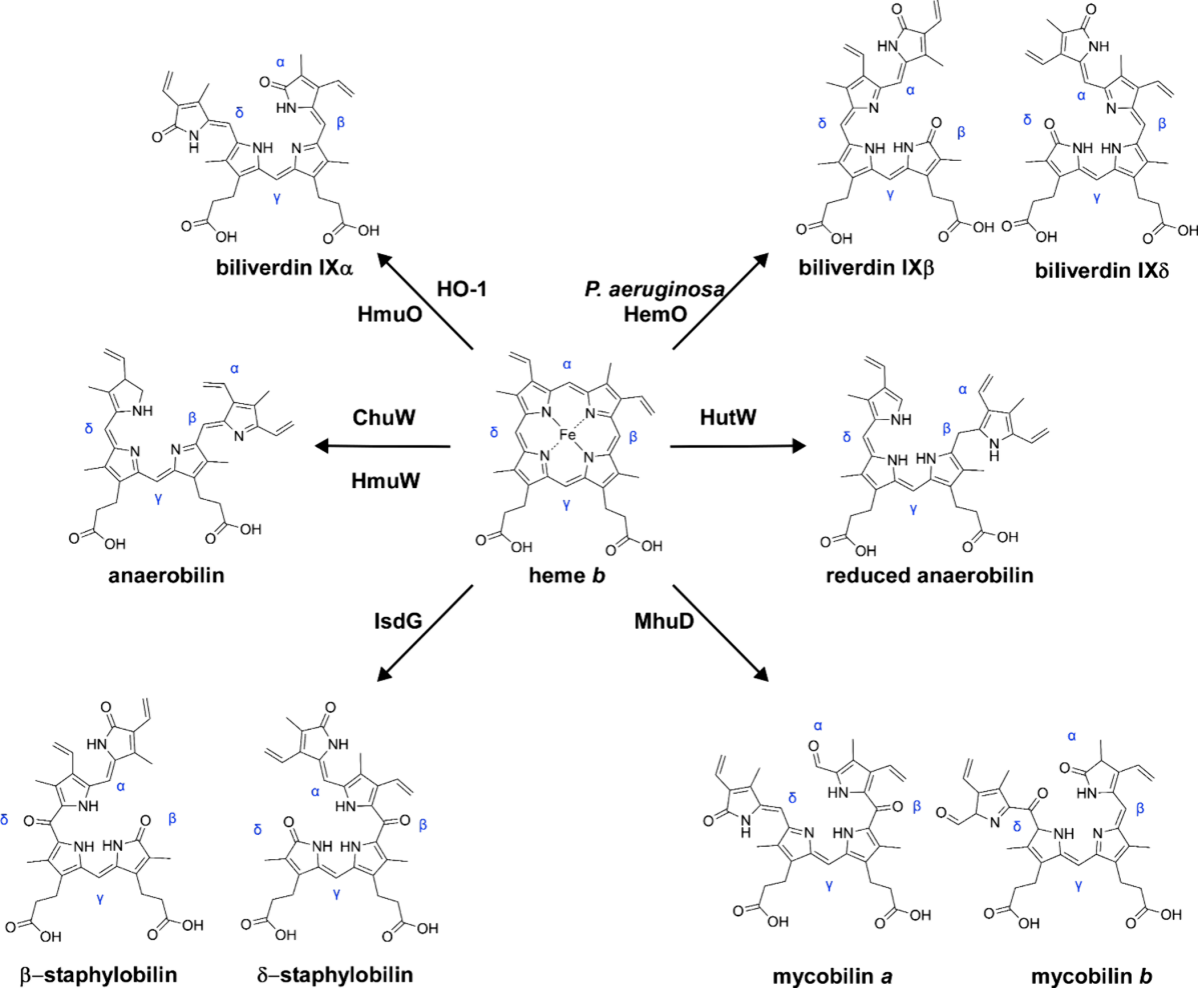
**Heme is metabolized to diverse chromophores by different degradation enzymes.** Heme *b* is catabolized by the canonical HO-1 family of heme oxygenases, IsdG-family heme oxygenases, IsdG-like MhuD heme oxygenase, and anaerobic heme degraders to diverse linear tetrapyrroles. Blue corresponds to *meso* carbons of heme *b*. Iron ion and single carbon byproducts, if applicable, are not shown.

### 5.1 HO-1 Family

The HO-1 family of heme oxygenases is known as the canonical heme oxygenases. This family of proteins is found in eukaryotes, Gram-negative bacteria, and some Gram-positive organisms.^248–254^ HO-1 heme oxygenases consist exclusively of α-helices, usually nine to ten, and their heme ligand sits in a planar orientation.^255^ They catalyze three steps of successive monooxygenation of heme to α-meso hydroxyheme, to α-verdoheme, then to oxo-biliverdin, and eventually, iron, BVIXα, and a single-carbon byproduct CO.^255–257^ The overall structures of the HO-1 family of proteins are highly conserved. Studies using a well-characterized apo-HO-1 from *Corynebacterium diphtheriae* ^250^ show that the proximal helix to the heme-binding pocket takes on a coiled or unwound structure.^258,259^ This leads to an upward kink in the distal helix, which also takes on a helix–coil–helix formation (Figure 3C). Together, the distal and proximal helices form an open structure in the pocket for the heme substrate.^258^ When heme is bound to the histidine residue on the proximal axis, this induces a conformational change to the proximal and distal helices, to form an organized, rigid “closed” pocket structure^258^ (Figure 3D). This closed conformation of the distal axis also provides a strong hydrogen bonding network for Fe-oxygen. As the distal helix sits on top of the heme ligand, it provides steric hindrance to the *meso* carbons on the tetrapyrrole molecule. As a result, oxygen is directed toward the α-*meso* carbon available to hydroxylation.^258^ HO-1 family of oxygenases commonly use NADPH-cytochrome P450 reductase to recycle their electrons.^260^ HO-1 heme oxygenases allow bacteria to use heme as an iron source, as seen in *Brucella abortus,* ^261^ *L. interrogans,* ^262^ *Neisseria gonorrheae* and *N. meningitidis* ^263^ to name a few.

#### 5.1.1 *Pseudomonas* HemO and BphO

*P. aeruginosa* harbors an interesting example of an HO-1 heme oxygenase. Although structurally similar to HemO of *Neisseria meningitidis*, *P. aeruginosa* HemO releases BVIXδ and BVIXβ in a 70:30 ratio.^225,264^ This is due to a 110° planar rotation of the heme ligand in the active site, exposing the β/δ-*meso* axis to facilitate hydroxylation.^265^ Further structural studies have revealed differences in residues determining heme positioning in *N. meningitidis* HemO, human HO, and *P. aeruginosa* HemO.^266^ The conserved heme propionate-interacting lysine and tyrosine in *N. meningitidis* HemO and human HMOX1 correspond to asparagine and phenylalanine in *P. aeruginosa* HemO. Mutation of these residues in *P. aeruginosa* HemO leads to the release of BVIXα as the major product,^267^ confirming the regioselectivity of heme positioning by the surrounding residues. A *P. aeruginosa* strain engineered to produce only the regioisomer BVIXα exhibits a proliferation defect, and shows reductions in motility, chemotaxis, and biofilm formation in a heme-dependent manner.^267^ Proteomic analysis of this strain further reveals that BVIXβ/δ may be signaling molecules participating in the quorum sensing system of *P. aeruginosa* during infection.^267^ PhuS, the cytoplasmic heme transporter, directly binds HemO to facilitate heme transfer through interactions with the charged residues of the heme pocket and conformational rearrangement in HemO.^268,269^ However, leaky transport of heme to HemO and a second heme oxygenase, BphO, can occur stochastically under high heme concentrations in the absence of PhuS.^270^ Deletion of *hemO* leads to less heme influx compared to wildtype strain, as measured by the lack of intracellular BVIXβ/δ in the Δ*hemO* strain.^270,271^ The regulation of heme metabolic flux is possibly through transcriptional or post-transcriptional regulation of *hasAp* mRNA based on BVIXβ and/or BVIXδ abundance,^221,269^ leading to decreased production of the hemophore. This highlights the feedback regulatory role of heme degradation in heme utilization.^270^

Although PhuS shares homology with HO-1s and can degrade heme,^228^ HemO is the dominant heme degradation enzyme; a Δ*phuS* mutant can still generate BVIXβ/δ.^225^ *P. aeruginosa* harbors another heme oxygenase, BphO, which releases BVIXα,^272^ consistent with canonical HO-1 family proteins. Unlike *hemO*, expression of *bphO* is not regulated by Fur, but instead is regulated by the quorum sensing regulator LasR and alternative σ factor RpoS during stationary growth phase.^273^ *bphO* is cotranscribed with *bphP*, encoding for a putative light-sensing phytochrome which presumably binds the heme degradation products of BphO, though it can also utilize BVIXδ *in vitro.* ^273^ Genes involved in the biosynthesis of *P. aeruginosa* quinolone signal precursors are downregulated in a Δ*bphO* mutant. Interestingly, under different ambient light exposure, PrrH sRNA levels are impacted.^241^ However, this phenotype is independent of BphO,^241^ therefore, the contribution of the photoreceptor BphP requires further investigation. Together, these results establish the potential role of heme catabolites as signaling molecules and underscore their complicated regulation mechanisms within bacterial systems.

### 5.2 IsdG Family

The noncanonical IsdG family is defined by its founding members IsdG and IsdI from *S. aureus* *.* ^210,211^ The IsdG family of heme oxygenases are structurally distinct from their HO-1 counterparts, in which they comprise of both α-helices and β-sheets, and the protomers dimerize at the sheets.^210^ Enzymatically, IsdG family proteins degrade heme to iron, β/δ-staphylobilin regioisomers, and formaldehyde.^212,274,275^ The heme ligand in IsdG family proteins is ruffled, where the heme-iron is bent toward the histidine residue at a 140° angle,^276,277^ as compared to the planar heme ligand in HO-1 proteins. This ruffling is essential for releasing staphylobilin as the product.^212,276,278^ IsdG proteins are postulated to convert heme sequentially to ferric peroxoheme, *meso*-hydroxyheme, ferric formyl staphylobilin, and finally the linear chromophore staphylobilin.^274,275,279,280^ The IsdG family is characterized by conserved catalytic residues, asparagine (Asn), tryptophan (Trp), and histidine (His). The Asn forms hydrogen bonds with the peroxo ligand of the intermediate ferric peroxoheme to stabilize the iron, making the *meso* carbons more electrophilic.^281,282^ The heme transition state stabilizing residue Trp contributes to the substrate distortion; mutating this Trp to a sterically less bulky residue allows the heme substrate to stochastically take on a planar conformation, and thus BVIXα can be produced.^278,283^ This Trp residue also stabilizes the immediate product following the first catalytic step of heme degradation.^279^

While *S. aureus* IsdG and IsdI can bind non-iron protoprophyrins and form stable complexes, the catalytic activities of these enzymes are specific for heme.^276^ Both *isdG* and *isdI* are Fur-regulated in *S. aureus* *.* ^284^ However, IsdG is post-translationally stabilized by heme, while IsdI is not.^284,285^ The flexible loop linking the helices and sheets within an IsdG monomer is required for protease-targeted degradation without heme, but it is not the proteolytic site.^286^ IsdI has a higher *K*_d_ value for the heme ligand than that of IsdG,^282^ which could point to a substrate-dependent regulation of enzymatic functions of these homologues. This differential regulation of heme oxygenases in *S. aureus* could serve as an alternative feedback mechanism, since IsdG inhibits CpfC, which catalyzes the last step in the CPD heme biosynthesis pathway.^287^ In a mouse systemic infection model, an Δ*isdG* mutant exhibits reduced bacterial burdens in the heart and kidneys compared to Δ*isdI,* ^285^ further supporting that these heme oxygenase paralogs play distinct roles during infection and are not functionally redundant.

IsdG and the IsdG-like family of heme oxygenases are reported in other Gram-positive organisms, such as *Staphylococcus lugdunensis,* ^288,289^ *B. anthracis,* ^290^ and even in eukaryotic organisms.^248^ Deleting *isdGI* in *S. aureus* confers a defect in colonization in a mouse systemic infection model.^285^ A *B. anthracis* mutant lacking one of its three IsdG-like proteins (Δ*hmoB*) is more susceptible to phagocytic killing compared to wildtype, but a mutant lacking all heme oxygenases exhibits significantly decreased bacterial burden in the lungs, heart, kidneys, spleen, and liver in a murine intranasal infection model.^291^ The evolutionary driving force that gave rise to diverse families of heme oxygenases remains elusive. There is a possibility that staphylobilin may function as feedback substrates or signaling molecules within these Gram-positive organisms, as seen with BVIXβ/δ in *P. aeruginosa*. Further studies of the functions of noncanonical heme catabolites may shed light on the regulatory network linking heme metabolism and virulence.

### 5.3 *Mycobacterium* MhuD

MhuD from *M. tuberculosis* represents a third class of heme oxygenases. Although previously thought to be an IsdG protein, MhuD generates a different chromophore, mycobilin, without generating a single-carbon byproduct.^292^ Mycobilin is characterized by breakage at the α-*meso* carbon, and a carbonyl group at the β- or δ-*meso* carbon.^293^ Despite sharing high sequence and structural homologies with *S. aureus* IsdG,^292^ MhuD can form both monoheme and diheme complexes.^294^ In the monoheme complex, MhuD catalyzes the degradation of heme through a monooxygenation step, where the heme molecule sits at a ruffled conformation, and sequentially a deoxygenation step, where the *meso*-hydroxyheme intermediate is in a planar conformation.^295–297^ Previously, the diheme complex of MhuD had been thought to be enzymatically inactive.^294^ However, mycobilin is still produced by diheme-MhuD complexes.^298^ This structural dynamic has led to two main hypotheses for the physiological functions of MhuD: one being that MhuD could serve as a heme sink and storage in high heme concentration,^299^ while the other being that the second heme in the diheme-MhuD may have a regulatory role in heme biosynthesis like IsdG of *S. aureus* *.* ^298^

### 5.4 Anaerobic Heme Degraders

In addition to detoxification by limiting influx and promoting efflux of excess heme, anaerobic heme degradation enzymes are increasingly being recognized for playing a role in heme detoxification. In the genomes of the hemorrhagic *E. coli* O157:H7 and other pathogenic strains lie the *chuAS chuTWXYUhmuV* heme utilization operons. ChuW is an anaerobic heme degradation enzyme that uses flavodoxin as an electron donor to generate S-adenosylmethionine radicals.^300^ The radical performs nucleophilic attack to ultimately target the α-*meso* carbon of heme and degrade it to anaerobilin.^301^ The [4Fe-4S] cluster of ChuW is readily inhibited by the presence of oxygen,^300^ shedding light on post-translational regulations of different heme degradation proteins within facultative anaerobes. ChuW binds heme, non-iron PPIX (cobalt and zinc), and PPIX.^300–302^ One report suggests that PPIX is likely not an intermediate product within the anaerobilin production reaction, but a *bona fide* demetalation product of ChuW.^302^

Downstream of *chuW* in the same operon is *chuY*. ChuY is an anaerobilin reductase that works in conjunction with ChuW.^303,304^ *Vibrio cholerae* has a similar, Fur-regulated, heme-induced operon, *hutWXZ.* ^305^ HutZ is a heme oxygenase;^230,306^ HutW is an anaerobic heme degrader whose mechanism is similar to ChuW, but differs in that it produces reduced anaerobilin.^307^ HutX transports heme to HutZ aerobically and to HutW anaerobically.^308,309^ Homologues of ChuW are also reported in *Fusobacterium nucleatum* ^310^ and the Gram-positive pathogen *E. faecalis.* ^311^ *E. faecalis* anaerobilin synthase OG1RF_RS05575 allows the bacterium to effectively utilize heme as a sole iron source in a mouse gut colonization model.^311^

ChuS is an HO-1 heme oxygenase harbored by commensal strains of *E. coli*. Like *P. aeruginosa* PhuS, ChuS can degrade heme under aerobic conditions.^228,301^ It was first reported that, under anaerobic conditions, ChuS only acts as a heme storage and transfers heme to ChuW for degradation.^301^ Another group later showed that ChuS and close homologues *Y. enterocolitica* HemS, *Y. pestis* HmuS, and *S. dysenteriae* ShuS, are NADH-dependent, anaerobic heme degraders *in vitro.* ^312–314^ This discovery is supported by the fact that these organisms are all facultative anaerobic pathogens. The heme degradation products of these enzymes have not yet been characterized, but they could represent a separate family of anaerobic heme degradation proteins if the products are not anaerobilin. These findings suggest that anaerobic heme catabolism is an indispensable, critical biochemical mechanism in Gram-negative and Gram-positive pathogens under hypoxia.

## 6 Therapeutics Based on Heme

The increasing prevalence of antibiotic resistance necessitates innovative approaches in antimicrobial drug development. The heme synthesis and uptake pathways in bacterial pathogens are often highly conserved and essential, making them attractive therapeutic targets. Here, three main classes of heme-based therapeutics are discussed (Figure 6), along with advancements in the effort to identify novel heme homeostasis participants as drug targets.

**Figure 6:**
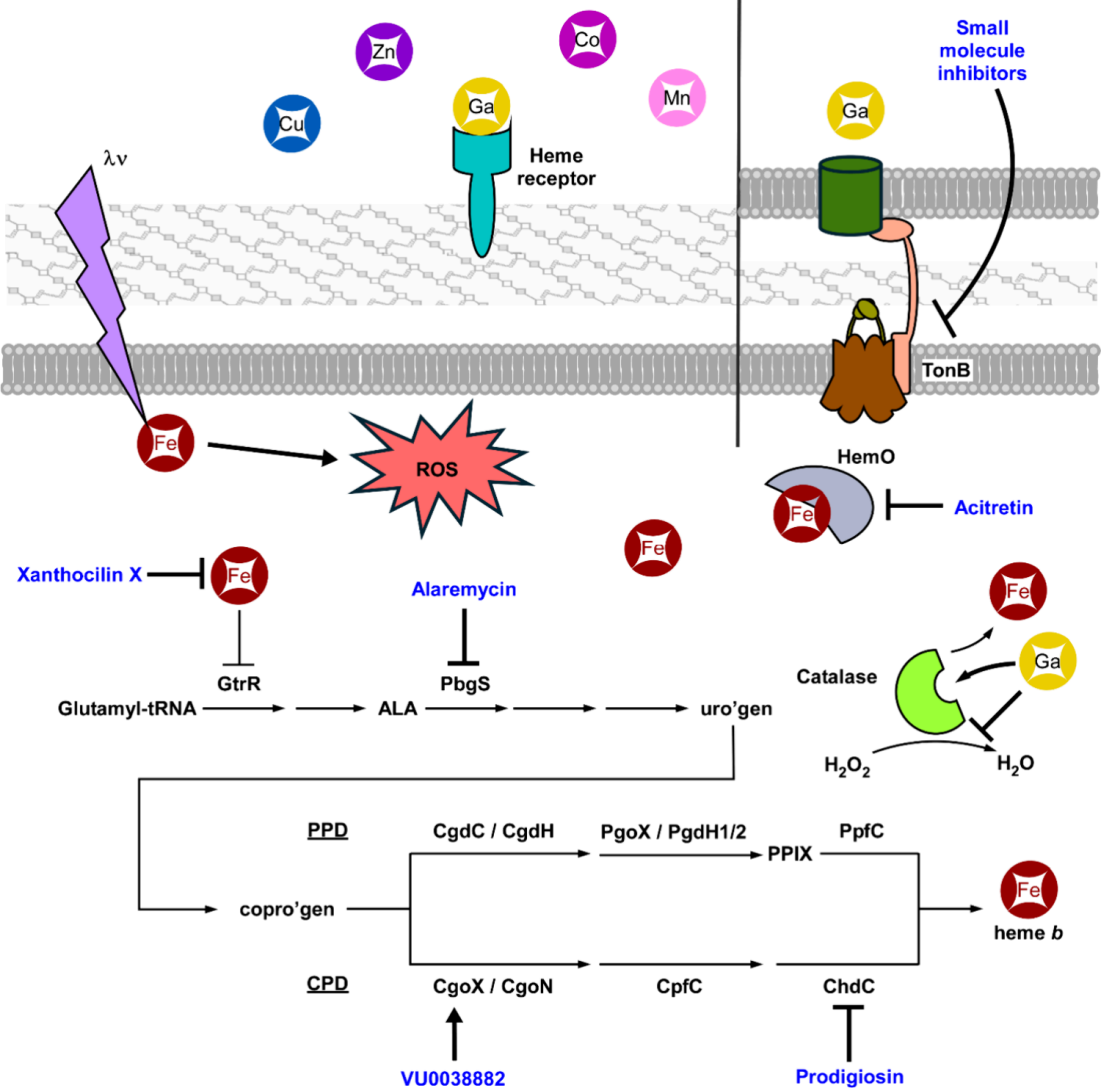
**Targeting the heme homeostasis pathways in bacteria.** Maintenance of heme homeostasis is crucial for invading pathogens, which makes the machineries involved in this process attractive targets for the development of novel, antimicrobial therapeutics. λν, photon energy, representing blue or green light, 405–480 or 522 nm. Circles with metal ions, metalloporphyrins; Ga, gallium; Cu, copper; Mn, manganese; Zn, zinc; Co, cobalt. PPD, protoporphyrin-dependent pathway; CPD, coproporphyrin-dependent pathway; uro’gen, uroporphyrinogen III; copro’gen, coproporphyrinogen III; ALA, 5-aminolevulinate acid; PPIX, protoporphyrin IX. In blue, drugs targeting heme homeostasis pathways.

### 6.1 Heme Mimics

PPIXs loaded with non-iron metals can be utilized as competitive inhibitors for bacterial heme acquisition. Gallium (Ga), a Group 13 metal, mimics ferric iron in terms of ionic radius and binding affinity for iron-binding proteins.^315–317^ However, gallium is redox inactive because Ga^3+^ is not reduced back to the Ga^2+^ state under physiological conditions.^318,319^ Therefore, Ga-PPIX can be used to deliver redox inactive metals in place of iron to disrupt redox cycling in bacterial cells. In a *S. aureus* model, ligand recognition domain NEAT3 on IsdH is unable to distinguish Ga-PPIX from ferric heme due to their similarities in three-dimensional structures and affinities for the receptor.^320^ Nanovesicles loaded with Ga-PPIX on their surfaces mimic erythrocytes for acquisition by *Porphyromonas gingivalis*, leading to a decrease in periodontitis in a rat model.^321^ Ga-PPIX has been demonstrated to inhibit growth of *S. aureus* *,* ^322–324^ *P. aeruginosa,* ^325–327^ *Acinetobacter baumannii,* ^328,329^ *Klebsiella pneumoniae,* ^330^ *M. tuberculosis* ^331^ *in vitro* or *in vivo*. Other non-iron, divalent metalloporphyrins also show antimicrobial activities by metal intoxication. Cobalt-PPIX and copper-PPIX effectively inhibit *P. gingivalis* growth in both planktonic and biofilm states, suggesting their efficacy in disrupting heme utilization pathways.^332,333^ Zinc-PPIX and manganese-PPIX inhibit *S. aureus* growth *in vitro.* ^322^ Administering cobalt-PPIX to *E. coli* decreases intracellular heme concentrations,^333^ indicating that heme mimics are recognized by the heme sensing system, leading to downregulation of heme intake mechanisms in pathogens.

Two major concerns regarding the use of iron-mimic metals are potential adverse effects to the mammalian hosts and acquisition of resistance to these treatments by bacteria, as bacteria may downregulate physiological processes that require iron as cofactors. Similar to bacterial heme and iron uptake through hemophores and siderophores, mammalian heme-sequestering immune proteins may also not be able to discriminate these “trojan horses”.^316,317,329^ One study showcases the efficacy of simultaneous, topical delivery of an iron chelator and Ga-PPIX in reducing *S. aureus* biofilm mass in a sheep rhinosinusitis model, while exhibiting no significant histopathological changes at the sinus mucosa.^334^ Another study reports Ga-PPIX topical delivery exhibits no cytotoxicity to human keratinocytes and fibroblasts *in vitro* and is effective against biofilm formation in a *S. aureus* mouse skin biofilm infection model.^335^ This combinatorial approach is demonstrated to reduce *S. aureus* small colony variants.^336^ However, emerging reports show that in the presence of sublethal gallium levels, *E. coli* and *S. aureus* may be able to adjust their metabolic landscape to tolerate Ga toxicity. These metabolic switches include increasing production of nonheme iron uptake machineries and upregulating damage responses, such as [Fe–S] cluster repair mechanisms.^293,337,338^ Development of metal-based antibacterial drugs may face the same challenges as those of conventional antibiotics.

### 6.2 Photodynamic Therapy

In conjunction with non-iron metalloporphyrin delivery, antimicrobial photodynamic therapy has shown low toxicity to the host while targeting microbial pathogens. Blue light exposure induces photosensitization of metalloporphyrins, which oxidizes labile heme-iron and heme to generate cytotoxic ROS.^339,340^ The bactericidal effects depend on the endogenous biosynthesis of heme and porphyrin intermediates. Blue light therapy reduces *P. aeruginosa* burden in a mouse skin infection model.^341^ *S. aureus* strains lacking GtrR are less sensitive to blue light treatment.^342^ Photoinactivation of hemoproteins, such as catalase, sensitizes bacteria to environmental assaults that would otherwise be detoxified by the hemoproteins.^343^ To augment the photoinhibitory efficacy of blue light therapy against heme-auxotrophic pathogens, such as *E. faecalis*, supplementation of exogenous ALA enhances growth inhibition *in vitro.* ^344,345^ A combination of metal intoxication and photosensitization demonstrates synergy as a novel therapeutic approach. A small molecule activator of CgoX increases endogenous heme production^346,347^ and an exogenous porphyrin-based, photosensitizer-conjugated monoclonal antibody, which targets *S. aureus* IsdA or IsdB, together reduced bacterial burden in a murine skin infection model.^348^ Aluminum-PPIX and Ga-PPIX administered to *S. aureus* inhibit planktonic growth upon exposure to blue light at 405 nm;^349,350^ Ga-porphyrins generate significant amount of ROS under green light (522 nm) activation.^350,351^ Synthetic photosensitizers that are either porphyrin-based or of similar properties are also being explored for their antibacterial efficacy.^350,352–354^

### 6.3 Disruption of Heme Homeostasis

Another critical area of research involves the inhibition of bacterial heme biosynthesis pathways. Motivated by the findings that mammalian, Gram-negative, and Gram-positive organisms harbor diverse heme biosynthesis enzymes and generate endogenous heme through different intermediates,^151,152,184^ the discovery of novel antimicrobials is beginning to target specific enzymes within these pathways. The mechanism of action of Xanthocillin X, an isonitrile class antibacterial compound, is to upregulate endogenous heme biosynthesis in bacterial pathogens, by forming a complex with free heme.^355^ The Xanthocillin-heme complex reduces the regulatory heme signal to dampen endogenous heme production by negative feedback, thereby causing an accumulation of oxidation-prone porphyrins intracellularly.^356^ Xanthocillin X is especially effective against multidrug-resistant *A. baumannii.* ^355,356^ Alaremycin, isolated from *Streptomyces sp.*, orthosterically displaces the universal heme precursor ALA in the porphobilinogen synthase (PbgS) active site, which leads to growth inhibition of *P. aeruginosa.* ^357,358^ Gallium-loaded salophen, a synthetic aromatic macromolecule, binds *P. aeruginosa* HasA­(p) at the heme-binding site and prevents downstream *has* operon signaling cascade.^359^ In addition to inhibiting transcription of heme uptake genes, Ga-salophen also subjects *P. aeruginosa* to gallium intoxication through the iron uptake system, thereby achieving growth inhibition *in vitro* by two mechanisms.^359^ Small molecule VU0038882 allosterically activates CgoX in Gram-positive pathogens, resulting in the overproduction and accumulation of endogenous heme intracellularly. VU0038882 is bactericidal in fermentative *S. aureus* *,* ^346^ but has low affinity for human protoporphyrinogen oxidase.^347^ A novel screen for drugs targeting the diverse heme biosynthesis enzymes, developed based on competitive viability assay between bacteria using terminal heme synthesis genes in the PPD or the CPD pathways,^360^ shows promise by preliminarily identifying tripyrrole molecules that selectively inhibit the growth of CPD enzyme-producing bacteria.^360^ This approach can be implemented in a high-throughput manner to screen existing molecule databases, facilitating repurposing of safety-certified compounds.

Inhibitory molecules targeting other parts of the bacterial heme utilization pathways are also being explored. Under an iron acquisition screen using a uropathogenic *E. coli*, inhibitors of TonB were identified.^361^ Although not yet demonstrated to directly affect heme acquisition in *E. coli*, these inhibitors presumably can work against TonB-dependent heme uptake systems in Gram-negative pathogens. *In silico* docking simulations highlight heme oxygenases as valuable potential drug targets. Low molecular weight compounds available from commercial vendors show inhibitory activities against *N. meningitidis* HemO^362^ and *P. aeruginosa* HemO.^362–365^ Acitretin, a retinoid, regioselectively occupies the *P. aeruginosa* HemO active site over those of other BVIXα-producing HO-1 proteins, such as human HOs,^366^ effectively rendering *P. aeruginosa* HemO unable to degrade heme. Proteomic assays to identify previously uncharacterized hemoproteins and proteins participating in heme homeostasis reveal potential drug targets.^367–370^ Improvements in computational prediction of molecular docking and interaction simulations, along with biochemical advancements, accelerate the high-throughput screening of compounds by simultaneously selecting for potential inhibitory activities and against off-target effects.

## 7 Concluding Remarks

Maintenance of heme homeostasis is imperative to proper survival and pathogenesis of bacterial pathogens that require heme. Heme sits at this pivotal axis in serving as a cofactor and an iron source to power biochemical reactions, which allow bacteria to proliferate, adapt, and evade host immune responses. The diverse strategies employed by bacteria to acquire, transport, utilize, and detoxify heme underscore the importance of heme homeostasis. Understanding the regulation and functions of these processes may shed light on their contributions to bacterial infections and highlight novel targets of therapeutic interventions.

Studies aiming to identify previously uncharacterized proteins involved in these pathways, to elucidate new functions of known hemoproteins, and to determine evolutionary functions of different classes of enzymes and catabolites could yield innovative approaches to combat bacterial infections. Expanding the understanding of bacterial heme homeostasis in the context of host–pathogen interaction is crucial to maximize antimicrobial effects, while minimizing toxicity to mammalian hosts. Targeting fundamentally conserved mechanisms marks an effective strategy to eradicate multidrug-resistant bacterial infections. This will greatly reduce the probability of pathogens acquiring resistance, but it does not rule out inducing tolerance. Combinatorial approaches using heme-based therapeutics are promising and, perhaps, will help launch the next generation of antimicrobial discovery.

## Abbreviations

HO-1: heme oxygenase 1
BVIXα: biliverdin IX alpha
CO: carbon monoxide
DAMP: damage-associated molecular pattern
TLR2: Toll-like receptor 2
TLR4: Toll-like receptor 4
poly I:C: polyinosinic-polycytidylic acid
LPS: lipopolysaccharides
IFNγ: interferon gamma
TGF-β: transforming growth factor beta
ROS: reactive oxygen species
RNS: reactive nitrogen species
MAPK: mitogen-activated protein kinase
PI3K/Akt: phosphoinositide 3-kinases/protein kinase B
NF-κB: nuclear factor kappa B
PPARα: peroxisome proliferator-activated receptor alpha
Fe: iron
NO: nitric oxide
PPIX: protoporphyrin IX
ALA: 5-aminolevulinate acid
uro’gen: uroporphyrinogen III
copro’gen: coproporphyrinogen III
PPD: protoporphyrin-dependent
CPD: coproporphyrin-dependent
NEAT: NEAr-iron transporter
NADPH: nicotinamide adenine dinucleotide phosphate
BVIXδ: biliverdin IX delta
BVIXβ: biliverdin IX beta
Asn: asparagine
Trp: tryptophan
His: histidine
Ga: gallium
Fe–S: iron–sulfur
HCHO: formaldehyde
